# Community-based diabetes prevention randomized controlled trial in refugees with depression: effects on metabolic outcomes and depression

**DOI:** 10.1038/s41598-023-35738-9

**Published:** 2023-05-30

**Authors:** Julie A. Wagner, Angela Bermúdez-Millán, Thomas E. Buckley, Orfeu M. Buxton, Richard S. Feinn, Sengly Kong, Theanvy Kuoch, Mary F. Scully

**Affiliations:** 1 University of Connecticut Schools of Medicine and Dental Medicine, UConn Health, 263 Farmington Ave., Farmington, CT 06030 USA; 2 University of Connecticut School of Medicine, Farmington, CT USA; 3 University of Connecticut School of Pharmacy, Storrs, CT USA; 4 Pennsylvania State University, State College, PA USA; 5 Quinnipiac University, Hamden, CT USA; 6 Khmer Health Advocates, West Hartford, CT USA

**Keywords:** Psychology, Diseases, Health care, Risk factors

## Abstract

Depression and antidepressant medications increase risk for type 2 diabetes. Cambodian-Americans have exceedingly high rates of both depression and diabetes. This paper reports outcomes of a diabetes prevention trial for Cambodian-Americans with depression. Primary outcomes were HbA1c, insulin resistance and depressive symptoms. Participants were aged 35–75, Khmer speaking, at risk for diabetes, and met study criteria for likely depression by either (a) antidepressant medication and/or (b) prolonged elevated depressive symptoms. Participants were randomized to one of three community health worker (CHW) interventions: (1) lifestyle intervention called *Eat, Walk, Sleep* (EWS), (2) EWS plus medication therapy management sessions with a pharmacist/CHW team to resolve drug therapy problems (EWS + MTM), or, (3) social services (SS; control). Assessments were at baseline, post-treatment (12 months), and follow-up (15 months). The n = 188 participants were 78% female, average age of 55 years, half had a household income < $20,000, and modal educational attainment was 7.0 years. Compared to the other arms, EWS + MTM showed a significant decrease in HbA1c and a trend for reduced inflammation and stress hormones. Depressive symptoms improved for EWS and EWS + MTM relative to SS. There was no change in insulin resistance. Cardiometabolic and mental health can be improved in tandem among immigrant and refugee groups.

## Introduction

Depression is associated with increased risk for type 2 diabetes, with relative risks (RR) ranging from 1.26 to 1.60^1,2^. Depression may increase risk for diabetes through behavioral pathways such as poor diet, physical activity, and sleep patterns. Depression-related stress hormones^3^ and inflammation^4^ may also contribute to metabolic risk. The use of antidepressant medications is also associated with increased risk for diabetes (RR = 1.68)^5^ with a relative risk that is slightly higher than untreated depression (RR = 1.56).

The Diabetes Prevention Program (DPP;^6^) showed that compared to the control condition, both a lifestyle intervention and metformin reduced the incidence of diabetes. Furthermore, it demonstrated that the lifestyle intervention was more effective than metformin in reducing incidence of diabetes. Lifestyle interventions for people with or at risk for diabetes can also reduce depressive symptoms in patients^7^. Yet, whereas there is some suggestion that individuals with depression^8^ and serious and persistent mental illness^9^ may benefit from diabetes prevention programs, there is a need for rigorous testing of diabetes prevention interventions for individuals with elevated symptoms of depression and/or those taking antidepressant medication. Individuals with depression may require unique or enhanced behavioral strategies for lifestyle change.

Cambodian refugees, who were resettled in the USA approximately 40 years ago fleeing the genocidal Pol Pot regime, have age- and sex-adjusted rates of type 2 diabetes more than double the US national average^10^. Rates of depression among Cambodian Americans are also alarming at 51%^11^. For many Cambodian Americans the depression becomes chronic, with pre-migration trauma increasing odds of major depressive disorder even decades after resettlement (odds ratio = 1.56)^11^. Cambodian Americans may receive medication for their health needs, but barriers related to language, finances, and very low educational attainment may create drug therapy problems with polypharmacy, drug safety, appropriateness, effectiveness, and adherence^12^. Medication therapy management (MTM) is a pharmacist intervention designed to resolve drug therapy problems^13^; MTM is not designed to test any particular medication.

Any diabetes prevention interventions for this group must take into account Cambodians’ significant barriers to lifestyle change including high rates of historic trauma^11^, high carbohydrate diet^14^, social isolation^15^ as well as their poor profile on the social determinants of health including high housing and food insecurity, low household income, and many have no formal schooling^16^. Barriers to lifestyle change that are specific to Cambodians include collective historic trauma^11^ which can impact emotional wellbeing, motivation, and problem solving, all of which facilitate behavior change. Cambodians have a history of starvation and their diet is high in carbohydrates^14^. Social determinants of health such as food insecurity and low educational attainment also pose barriers to healthy lifestyle^15,16^. Unfortunately, culturally adapted diabetes prevention programs often do not address these characteristics and so have suffered from low reach and engagement of minority populations^17^. Carefully selected, trained and supervised lay health workers may be in the best position to use culturally derived programs to reach and engage these hard-to-reach populations.

This paper reports the outcomes of a randomized, controlled trial of culturally derived diabetes prevention interventions for Cambodian Americans with depression, i.e., those with elevated symptoms of depression and/or taking antidepressant medication. We compared three arms: lifestyle vs lifestyle plus medication therapy management vs social services. Given the aforementioned evidence regarding the role of lifestyle in both glycemic status and depression, we hypothesized that, relative to social services, participants receiving lifestyle intervention would show improved depressive symptoms, HbA1c, and insulin resistance at post-intervention. Further, given evidence regarding the role of psychoactive medication in both glycemic status and depression, we hypothesized that the combined arm (lifestyle plus medication therapy management) would show more improvement in these primary outcomes than the lifestyle-only arm. In secondary analyses we examined effects on inflammation and stress hormones.

## Materials and methods

### Overview

*Eat, Walk, Sleep* (EWS) is a cardiometabolic lifestyle curriculum that was created through community based, participatory methods. It was created by and for Khmer people and is intended to be delivered by lay health workers^18^. We compared the efficacy of *Eat, Walk, Sleep* (EWS) vs. EWS plus medication therapy management (EWS + MTM) vs social services (SS, control condition). Assessments were at baseline, post-treatment (12 months) and follow-up (15 months). Study personnel who had contact with participants had been born in Cambodia and were bilingual and bicultural. To minimize bias, lay health workers were divided into two roles. Community health *educators* (CHEs) delivered intervention sessions. Community health *workers* (CHWs) conducted all data collection. Details of the lifestyle intervention and study protocol^19^ have been described previously. ClinicalTrials.gov identifier: NCT02502929 (20/07/2015).

### Subjects

The study was conducted according to the World Medical Association Declaration of Helsinki and approved by the UConn Health institutional review board. Participants signed written informed consent forms in their preferred language (Khmer or English), provided a release of information for study staff contact with their healthcare provider, and provided written HIPAA authorization. Participants were recruited through community and clinical settings.

Because this was a diabetes prevention intervention, individuals with extant diabetes were not eligible to participate. Inclusion criteria were: (1) aged 35–75; (2) Cambodian or Cambodian-American; (3) Khmer speaking; (4) currently living in Connecticut, Massachusetts, or Rhode Island; (5) lived in Cambodia during the Pol Pot regime (1975–1979); (6) ambulatory; (7) consumed meals by mouth; (8) elevated risk for diabetes per a modified version of the American Diabetes Association Risk Test^19^. Total risk score, rather than a single indicator such as HbA1c, was used for eligibility. The scoring included east Asian cutoffs for waist circumference as a measure of adiposity rather than BMI. Participants were also required to meet criteria for depression by (a) current antidepressant medication, and/or, (b) elevated depressive symptoms indicative of likely major depressive disorder on the Khmer language Hopkins Symptom Checklist^20^ with elevated symptoms on two occasions that were two weeks apart during a study screening and eligibility period. Exclusion criteria were: type 2 diabetes; seeing or hearing problems that would interfere with group sessions; major medical problems requiring intensive treatment; pregnancy or planning pregnancy; serious thinking or memory problems (e.g., schizophrenia or dementia); and 3 or more days in a psychiatric hospital or self-harm in the past 2 years.

As previously described^19^, power analysis based on published standard deviations of insulin resistance (logHOMA-IR) indicated the need for 210 participants with an allocation ratio of 1:1:1 (EWS:EWS + MTM:SS). In March 2016 recruitment began but by early 2017 it became apparent that the enrollment goal would not be met within the timeframe of the funding period. The standard deviation of logHOMA-IR for the 40 participants who had been randomized up to that point was used to conduct a revised power analysis which showed 80% power to detect treatment effects on primary outcomes with 175 completers at 12 months. Therefore, target sample size was reduced and the allocation ratio was changed to 2:2:1 going forward in order to gather relatively more information on the two intervention groups. The plan was approved by the funder and all other aspects of randomization remained the same.

### Assessments

CHW data collectors conducted assessments. Assessments were conducted in a private setting at a location of the participant’s choice, either in-home or at a clinic or social service agency. The CHWs administered surveys verbally and recorded responses in Remote Electronic Data Capture (REDCap)^21^ using a tablet. They also collected hair samples for the assessment of cortisol. Participants were compensated $10. On a separate day, participants presented to a nearby Quest Diagnostics to provide a fasting blood sample and were compensated an additional $10. Recruitment began in March 2016 and data collection ended in September 2020.

### Randomization

After baseline assessments, participants were individually randomized by a monolingual English speaking research assistant at UConn Health who had no direct contact with participants using an urn randomization^22^ computer program that balanced the three treatment arms on gender, age, symptoms of post-traumatic stress disorder, and site (Connecticut, Rhode Island, Massachusetts). The research assistant reported the participant’s assignment to the bilingual study coordinator who then notified the participant in Khmer. The study coordinator also telephoned to notify the site-specific CHE (interventionist) of the allocation. The CHWs, who conducted all study assessments, worked in close proximity to the CHEs so it was not possible to blind CHWs to allocation.

### Interventions

#### Eat, Walk, Sleep

EWS is a trauma-informed, cardiometabolic education curriculum based on Buddhist concepts of health and disease that is designed for delivery by CHEs to low literacy, low numeracy learners^18,23^. Behavioral targets of EWS include eating no more than 1 small bowl of (brown) rice per meal, walking at least 30 min per day on 6 days per week, and getting 7–9 h of restful sleep per night^24^. Those targets are worked toward over time using session-to-session goal setting.

Participants who were randomized to EWS or EWS + MTM were assigned to receive 3 individual EWS sessions and 24 group EWS sessions. They also received feedback/recommendation sessions at baseline and after their post (12 month) assessment during which laboratory results were presented and explained to participants along with lifestyle recommendations. The EWS lifestyle curriculum was designed to meet or exceed the published guidelines for diabetes prevention interventions set forth in the National Institute for Health and Clinical Excellence (NICE)^25^ and Implementation of A European Guideline (IMAGE)^26^.

#### Medication Therapy Management

MTM followed guidelines of the American Pharmacists Association^27^. The CHE, participant, and pharmacist identified drug-therapy problems. Problems were categorized as safety (does the patient have, or is the patient at risk for, adverse drug reactions?), appropriateness (is the medication indicated for the condition in this particular patient?), effectiveness (is the medication meeting clinical target?), and adherence (is the patient taking the medication as prescribed?). The CHE, participant, and pharmacist also created a medication action plan and sent a detailed report to the patient’s provider. This was not a drug trial. Participants who received MTM received consultations with a pharmacist to resolve any medication problems, but no specific medications were tested as part of the protocol. Patients and CHEs were together face-to-face and communicated with the pharmacist via telemedicine at least three times over the course of the 12 month intervention, and once again for a ‘booster’ session between 12 and 15 months. The four pharmacists were either nationally certified in MTM or board-certified in ambulatory care, geriatric, or psychiatric pharmacy.

#### Social services

Participants who were assigned to SS were assessed for any social service needs such as food or housing assistance, referral to a healthcare provider, tax preparation, or citizenship applications. CHWs were tasked with following up to meet any identified needs, and contacts were documented over the following 12 months.

### Measures

#### Demographics

Demographics included self-reported sex, age, income, employment, education, health insurance status and type, years in the U.S.

### Main outcomes

#### HbA1c

Glycosylated hemoglobin A1c (HbA1c) was assayed at Quest laboratory using direct enzymatic assay. Hemoglobinopathies, which vary by population, can make some HbA1c assays unreliable. Hb E is the hemoglobinopathy of most concern to this study because it is not uncommon in Southeast Asians. Therefore, we used a direct enzymatic assay because this method is unaffected analytically by Hb variants^28^.

#### Insulin resistance

Log transformed homeostatic model assessment of IR (logHOMA-IR) was calculated from fasting glucose and insulin values according to the standard formula^29^: log(fasting glucose x fasting insulin)/405.

#### Depressive symptoms

Symptoms of depression were assessed with the 15-item depression subscale of the Khmer language Hopkins Symptom Checklist using the published cutoff of mean = 1.75 (equivalent to sum = 26)^20^ to determine likely major depressive disorder. Cronbach’s alpha in this study was 0.93.

### Other variables of interest

#### Hair cortisol

Cortisol in human hair is a putative biomarker of chronic stress. Hair samples were obtained from approximately 2 cm below the cranial bone. Hair (1 cm, ~ 1 month of growth) was processed according to standard methods^30^: washed in isopropanol, dried, extracted on methanol and assayed using a standard ELISA kit.

#### High sensitivity C-reactive protein (hsCRP)

hsCRP is an inflammatory marker that is found to be high in the setting of insulin resistance and depression. hsCRP was measured by Quest Laboratories using an immunoturbidimetric assay.

For descriptive purposes only we also assessed lipids and anthropometrics over time. High density lipoprotein (HDL), low density lipoprotein (LDL), triglycerides (TGL), and total cholesterol were measured in mg/dL by Quest Laboratories with a spectrophotometry assay from Beckman Coulter. LDL was calculated using the Martin–Hopkins calculation^31^. Weight was measured using a calibrated electronic Seca (Chino, California) digital scale. Height was measured with the Seca portable stadiometer, with the participant’s head positioned in the Frankfurt horizontal plane. Waist circumference was measured at the umbilicus with an inelastic tape. Blood pressure was measured twice with calibrated digital sphygmomanometer (Omron, Hoffman Estates, IL)^32^. For all measurements, discrepant values of two trials exceeding a predetermined allowance triggered a third measurement and the two closest values were averaged.

### Statistical analysis

Linear mixed models (LMM) was used to compare groups at 12 month and 15 month time points on all main outcomes (HbA1c, insulin resistance, depressive symptoms) and other variables of interest (cortisol, hsCRP, waist circumference, BMI, systolic & diastolic BP, LDL, HDL, cholesterol, triglycerides). Fixed effects included group, time point, and the interaction between group and time point. To account for the correlation in the outcome values across time points the covariance was modeled using AR(1) with heterogenous variance and also a random intercept that varied by patient. Linear contrasts comparing the control group to the EWS and the EWS + MTM groups was performed on the change from baseline values at each time point. As a measure of effect size the test statistic from the linear contrast along with the degrees of freedom was used to calculate Cohen’s d^33^. All eligible participants were included in each analysis by original assigned groups. Alpha was set at 0.05 and analyses were conducted using SPSS v27.

### Human rights

All procedures performed in studies involving human participants were in accordance with the ethical standards of the institutional and/or national research committee and with the 1964 Helsinki declaration and its later amendments or comparable ethical standards.

## Results

**Figure 1:**
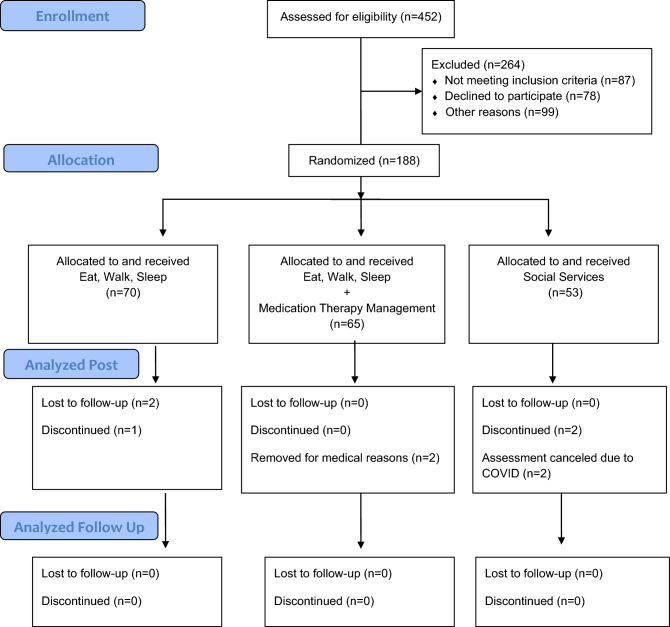
CONSORT diagram.

See Fig. 1 for Consort diagram; 188 participants were randomized. There were no significant differences among the three treatment groups in any of the baseline demographic or clinical characteristics (Table 1). The sample was 78% female, average age of 55 years, nearly 50% had a household income below $20,000, most (64%) were not working, and the average educational attainment was 7.0 years. They were on average 16 years old in 1979 at the end of the 4-year Pol Pot regime. At baseline, over half (54%) had elevated depressive symptoms and about one-third were taking antidepressant medication.

**Table 1:** Baseline characteristics (n = 188).

Characteristic	SS (n = 53)	EWS (n = 70)	EWS + MTM (n = 65)	P-value*
Gender				.220
Female	44 (83%)	50 (71%)	53 (82%)	
Age				.578
Mean ± SD	54.2 ± 8.5	55.9 ± 9.3	55.3 ± 8.5	
Household income				.890
Under $20,000	25 (53%)	30 (47%)	27 (48%)	
$20,000–30,000	9 (19%)	16 (25%)	16 (29%)	
$31,000–40,000	3 (6%)	7 (11%)	7 (13%)	
Over $40,000	10 (21%)	11 (17%)	16 (11%)	
Employment status				.633
Part/full-time working	22 (42%)	24 (34%)	22 (34%)	
Years education				.336
Mean ± SD	7.3 ± 5.1	7.4 ± 4.9	6.2 ± 5.1	
Health insurance				.755
Medicare	11 (22%)	17 (25%)	17 (27%)	
Medicaid	19 (37%)	23 (34%)	20 (32%)	
Private	17 (33%)	26 (39%)	22 (36%)	
Other	4 (8%)	1 (2%)	3 (5%)	
Years in US				.060
Mean ± SD	28.4 ± 10.7	25.6 ± 12.4	30.0 ± 10.9	
Depression inclusion criteria				
Taking antidepressant	16 (30%)	23 (33%)	24 (37%)	.735
Elevated symptoms	26 (50%)	39 (56%)	36 (56%)	.764

*SS* social services, *EWS* Eat, Walk, Sleep, *EWS* + *MTM* Eat, Walk, Sleep plus Medication Therapy Management.

*Chi-square for categorical variables, Mann–Whitney U for ordinal, ANOVA for continuous.

As has been reported elsewhere^19^, recruitment, attendance and retention were excellent, with 96% retention at 15 months. Of those randomized to EWS, 86% completed >  = 24 EWS sessions. Of those randomized to EWS + MTM, 77% also completed >  = 4 MTM sessions^19^.

Mean drug therapy problems per participant in the MTM arm at baseline was 6.6 (SD = 4.3)^13^. The most frequent type of drug therapy problem was safety (M = 2.6, SD = 2.8) and the least frequent type was effectiveness (M = 0.7, SD = 0.9). Drug therapy problems related to medications that are known to affect HbA1c were most frequent for statins (n = 15), selective serotonin reuptake inhibitors (SSRIs; n = 13), antipsychotics/antidepressants (n = 10), beta blockers (n = 6), thiazide diuretics (n = 5 and steroids (n = 4). Nearly 84% of all drug therapy problems were resolved, ranging from 74% resolution for effectiveness to 94% resolution for adherence. Most (88%) were resolved by the CHW-pharmacist team without intervention from the prescriber^13^.

**Figure 2:**
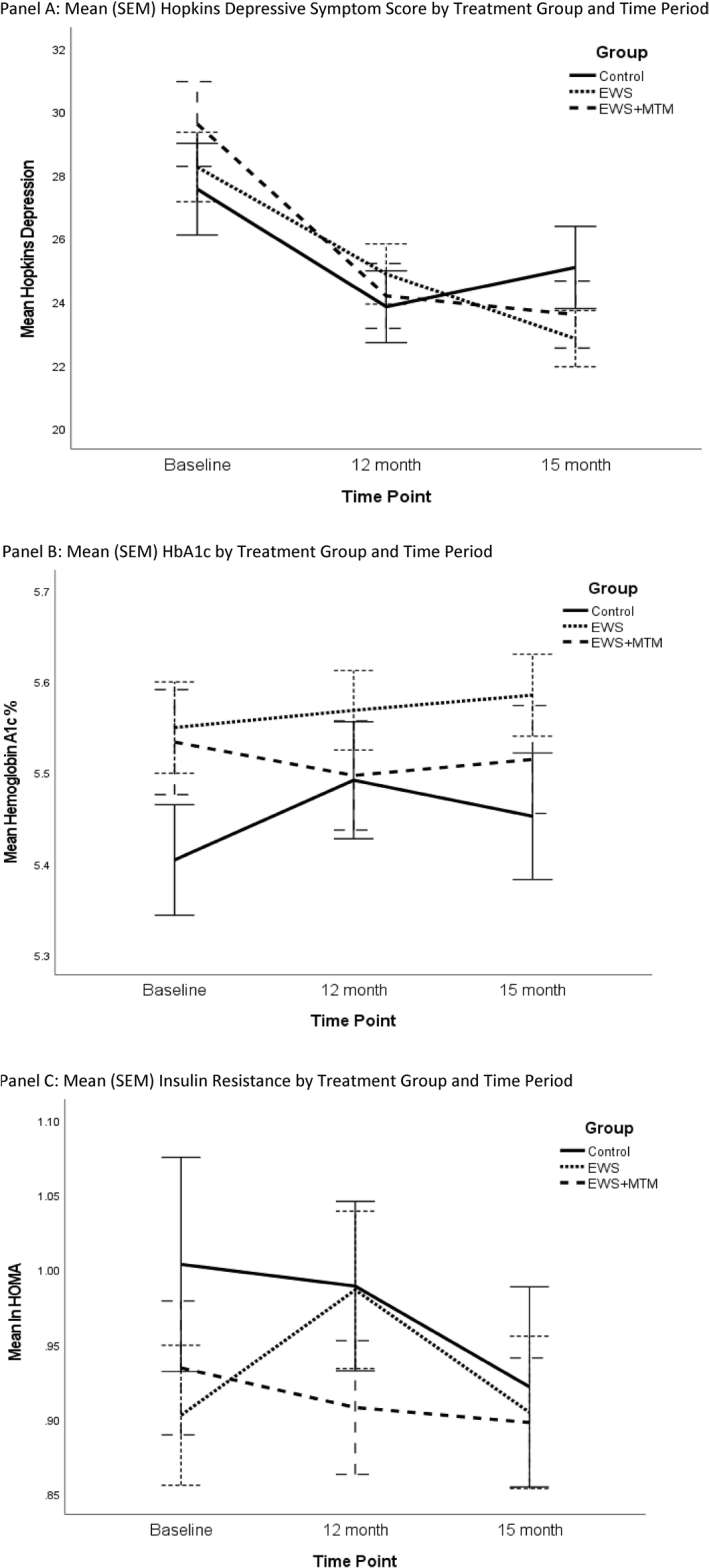
Changes in primary outcomes over time by arm.

Figure 2A shows Hopkins depression score across time points by treatment group and the top of Table 2 presents the means. There was a significant time point effect (Means: baseline  = 28.2, 12 month = 23.9, 15 month = 23.5, p < 0.001) such that all groups decreased from baseline to 12 month endpoint (p < 0.001) with no significant differences between 12 month and 15 month follow-up (p = 0.37). Contrasts revealed the change from baseline to endpoint did not differ between groups, but the change between baseline and follow-up did. Compared to the SS group, the EWS group had a greater reduction at follow-up (p = 0.043, Cohen’s d = 0.22) and likewise the EWS + MTM group had a greater reduction (p = 0.011, d = 0.27), while the two intervention arms did not differ from each other (p = 0.71, d = 0.06).

**Table 2:** Characteristics of each group by timepoint.

Characteristic	Baseline	12 Month	15 Month	P-value	P-value
				12 Month	15 Month
**Panel A: Primary outcomes means (± SD) by time point**					
**Depression**					
SS	27.5 ± 10.4	24.5 ± 7.9	25.5 ± 9.1		
EWS	28.2 ± 9.2	25.1 ± 7.8	23.1 ± 7.3	.785	.043
EWS + MTM	29.6 ± 10.7	23.6 ± 8.1	23.0 ± 8.4	.184	.011
**HbA1c %**					
SS	5.40 ± 0.43	5.57 ± 0.44	5.53 ± 0.49		
EWS	5.55 ± 0.41	5.53 ± 0.35	5.55 ± 0.37	.154	.757
EWS + MTM	5.53 ± 0.46	5.47 ± 0.47	5.50 ± 0.47	.011	.189
**Insulin resistance**					
SS	1.00 ± 0.52	0.97 ± 0.39	0.89 ± 0.47		
EWS	0.90 ± 0.39	1.00 ± 0.43	0.93 ± 0.41	.221	.197
EWS + MTM	0.93 ± 0.36	0.91 ± 0.35	0.90 ± 0.34	.689	.544
**Panel B: Other variables means (± SD) by time point**					
**Hair cortisol**					
SS	2.53 ± 1.39	2.99 ± 1.33	2.99 ± 1.95		
EWS	2.62 ± 1.13	2.77 ± 0.99	2.64 ± 1.29	.257	.176
EWS + MTM	2.64 ± 1.25	2.49 ± 1.18	2.49 ± 1.29	.051	.062
**hsCRP**					
SS	0.86 ± 0.60	0.93 ± 0.61	0.84 ± 0.55		
EWS	0.93 ± 0.63	0.83 ± 0.62	0.85 ± 0.57	.179	.825
EWS + MTM	1.05 ± 0.67	0.85 ± 0.55	0.89 ± 0.47	.096	.671
**Waist circumference**					
SS	92.0 ± 17.3	88.4 ± 10.3	87.5 ± 10.3		
EWS	89.1 ± 10.4	89.0 ± 10.6	89.0 ± 12.3	.306	.119
EWS + MTM	91.8 ± 10.1	88.9 ± 8.5	87.0 ± 8.4	.910	.536
**Body mass index**					
SS	26.6 ± 4.3	26.8 ± 3.7	26.5 ± 5.3		
EWS	27.2 ± 4.8	26.6 ± 4.5	26.2 ± 4.3	.556	.441
EWS + MTM	27.0 ± 3.2	26.5 ± 4.7	26.8 ± 3.5	.495	.665
**Systolic BP**					
SS	123 ± 18.4	123 ± 15.5	122 ± 16.0		
EWS	127 ± 21.9	124 ± 18.8	119 ± 15.4	.582	.115
EWS + MTM	127 ± 19.4	122 ± 15.8	120 ± 14.2	.261	.239
**Diastolic BP**					
SS	80.7 ± 10.0	78.6 ± 11.1	76.0 ± 9.2		
EWS	80.8 ± 11.5	78.3 ± 10.1	76.5 ± 9.4	.872	.860
EWS + MTM	81.4 ± 10.9	79.2 ± 8.2	76.1 ± 8.6	.906	.836
**LDL**					
SS	112 ± 32.9	116 ± 30.9	112 ± 29.1		
EWS	119 ± 31.1	115 ± 34.2	114 ± 32.3	.459	.907
EWS + MTM	115 ± 34.2	116 ± 34.6	113 ± 32.6	.794	.981
**HDL**					
SS	53.7 ± 14.4	55.2 ± 13.5	53.7 ± 12.0		
EWS	52.3 ± 17.5	53.9 ± 15.4	52.4 ± 14.6	.605	.672
EWS + MTM	52.8 ± 14.4	55.5 ± 13.8	55.6 ± 16.1	.757	.610
**Total cholesterol**					
SS	191 ± 37.8	195 ± 36.1	191 ± 33.2		
EWS	196 ± 33.7	193 ± 41.0	192 ± 37.7	.502	.932
EWS + MTM	196 ± 36.5	194 ± 40.9	191 ± 37.0	.644	.828
**Triglycerides**					
SS	142 ± 69.1	133 ± 54.6	143 ± 105		
EWS	129 ± 56.3	136 ± 69.7	137 ± 83.8	.490	.973
EWS + MTM	147 ± 85.1	124 ± 73.7	127 ± 64.5	.478	.173

The p-values for 12 month and 15 month are testing the specific intervention group (EWS or EWS + MTM) versus the control group, consistent with the a priori data analysis plan.

*SS* social services, *EWS* Eat, Walk, Sleep, *EWS* + *MTM* Eat, Walk, Sleep plus Medication Therapy Management, *BP* blood pressure, *LDL* low density lipoprotein, *HDL* high density lipoprotein, *hsCRP* high sensitivity c-reactive protein.

Figure 2B shows the mean profile across time points by group of HbA1c and the top of Table 2 presents the means. There was not a significant effect for time point (Means: baseline = 5.50, 12 month = 5.51, 15 month = 5.52, p = 0.59), however there was a significant difference in the change from baseline to 12 month between SS and EWS + MTM group (p = 0.011, d = 0.28), but not between SS and EWS (p = 0.154, d = 0.15). There were no significant differences between groups at 15 months.

Figure 2C shows the mean profile of the third primary outcome, insulin resistance (natural log transformed HOMA) and the top of Table 2 displays the means. There was no effect for time point (Means: baseline = 0.95, 12 month = 0.96, 15 month = 0.91, p = 0.14) and no significant differences between groups at the 12 month or 15 month time points.

The bottom of Table 2 displays the means for other biological variables for each treatment group by time point, along with the p-value from the tests comparing the two intervention groups versus the SS group on the change from baseline values. While there was a significant time point effect for waist circumference (p < 0.001), diastolic blood pressure (p < 0.001), and systolic blood pressure (p < 0.001) such that patients improved at 12 month compared to baseline and maintained the improvement at 15 month, the change from baseline did not differ between groups. There was no significant time point effects or group effects for BMI, LDL, HDL, cholesterol, triglyceride, or hsCRP. There were trends for the EWS + MTM group to show a decline in hair cortisol at 12 months (p = 0.05, d = 0.24) and 15 months (p = 0.06, d = 0.23) relative to controls. There was also a trend for the EWS + MTM group to show a decline in hsCRP at 12 months (p < 0.10, d = 0.18).

## Discussion

The findings for the primary outcomes are that (1) the EWS + MTM intervention decreased HbA1c relative to SS at post-treatment, (2) both treatment groups decreased depressive symptoms relative to SS at 15 months, and, (3) there was no change in insulin resistance. For other biological variables there were trends for the EWS + MTM group to show decreased inflammation at post-treatment and decreased stress hormones at post-treatment and at follow-up. Whereas they were not among our primary or secondary outcomes, we found that waist circumference, SBP and DBP decreased at post-treatment and follow-up for all groups. There were no significant changes in lipids.

Our first main finding is that in this sample, the combination of lifestyle and MTM reduced HbA1c, but lifestyle alone did not. The combination arm achieved a glycemia lowering effect of small-to-medium size (d = 0.28). This is commensurate with a meta-analysis of 69 lifestyle interventions for diabetes prevention at 12 months^34^. Whereas the effects are similar in size, our findings are compelling given that our participants were taking anti-depressants or had elevated depression symptoms, they had high rates of trauma exposure and post-traumatic stress, very low educational attainment, and our interventionists were CHEs. Also, whereas our participants had accumulated risk factors for diabetes, many had a normal baseline HbA1c, which created floor effects for some participants, making change in HbA1c harder to detect. Despite this, EWS + MTM produced HbA1c lowering effects on par with other diabetes prevention studies in populations that are ‘easier’ to treat.

It is important to note that the EWS + MTM group did not achieve superior glycemia via exposure to metformin. MTM recommendations to healthcare providers included the initiation of metformin for n = 16 of participants assigned to EWS + MTM—but none of them actually took or initiated metformin or other glucose lowering drugs. Indeed, we had hoped that, upon receiving the recommendation from the pharmacist accompanied by a detailed laboratory report, providers would initiate metformin, but in no case did this occur. Therefore, any effects of MTM were due to changes in other medications or to participant medication taking behaviors. Elsewhere we have reported that 90% of this sample had at least one drug therapy problem at baseline, the most common of which were safety problems. Our MTM intervention resolved 84% of those drug therapy problems^13^ including problems with safety, appropriateness, effectiveness, and adherence. We suspect that MTM mitigated drug interactions, optimized medication doses, and increased adherence. MTM provides pharmacists the opportunity to make salutary modifications to various medications that might affect HbA1c. For example, at baseline one participant had extant drug therapy problems with seven drugs that are known to effect HbA1c (albuterol [bronchodilator beta agonist], hydrochlorothiazide [thiazide diuretic], risperidone [atypical antipsychotic], trazadone [antidepressant], fluoxetine [serotonin-specific reuptake inhibitor], and fluticasone and dulera [both steroids]). Resolution of any of these drug therapy problems (increase/decrease dose, add/delete medication, switch to a different medication, or take as directed) may have had salutary effects on HbA1c.

It is important to remember that all participants met study criteria for depression at baseline. MTM provides the opportunity to resolve problems with psychiatric medications such as anti-depressants and third-generation antipsychotics that are associated with dysglycemia. Antidepressants are associated with an elevated risk of diabetes, with pooled adjusted Hazard Ratios (HRs) from 1.10 to 1.26 (e.g.,^35^). In the DPP, baseline antidepressant use was associated with conversion to diabetes in the placebo (HR 2.25) and lifestyle arms (HR 3.48). Among DPP participants who lost weight, those on antidepressants had higher risk of weight regain^36^. Pharmacists working with CHEs may be best positioned to make recommendations about balancing effective depression treatment with potential deleterious metabolic effects of antidepressant medications. We note that the decrease in HbA1c in the EWS + MTM group was not maintained at follow-up, suggesting that ongoing monitoring of medications and drug related problems may be necessary for high-risk populations like the one in this study.

Another main finding, that there was no change in insulin resistance, suggests that any beneficial effects of MTM on HbA1c were not mediated by increased insulin sensitivity. Whereas some medications may increase glycemia directly (e.g., steroids), others do so indirectly via their action on insulin sensitivity (e.g., second-generation antipsychotics). We speculate that the decrease in HbA1c in the EWS + MTM group without a concomitant decrease in insulin resistance suggests that any medication changes made by the pharmacist may have had direct benefits on glycemia.

The third main finding is that both treatment groups lowered depressive symptoms relative to SS, even though EWS was not designed as a depression treatment. EWS included scheduling, goal setting and increased socialization which are components of behavioral activation, a well validated depression treatment^37^ that can be delivered by lay health workers. EWS also included physical activity sessions which can have direct mood enhancing effects^38^. The SS arm did see a reduction in depressive symptoms; we suspect that this reduction was due to non-specific intervention factors often seen in behavioral studies (such as attention which creates the very rationale for an attention-control condition). However, the decrease in depressive symptoms from baseline to follow-up was greater for the EWS and EWS + MTM groups than for the SS group. This demonstrates that the active interventions had a stronger depression treatment effect than the SS comparison group.

Surprisingly, all groups showed decreased blood pressure and waist circumference over time. Whereas the SS group did not receive either of our active interventions per se, they did receive on-demand social services from CHWs that could putatively affect health such as referrals for food assistance, healthcare providers, assistance processing insurance documents, and translation of written materials. This finding underscores the health benefits of addressing ‘upstream’ social determinants of health, especially in underserved populations. Alternatively, contamination may have contributed to the benefits observed in the SS group. We ensured that people from the same family and/or household were randomized to the same arm. Nonetheless, since this is a tight-knit community, participants who were randomized to treatment may have shared intervention information with their friends from different families/households who were assigned to the SS group.

That waist circumference (but not BMI) decreased over time for all groups may reflect that glycemia of east Asians, relative to other groups, is more sensitive to *central* adiposity. It has been consistently observed that diabetes presents at a lower BMI in east Asians compared to whites. For any given BMI, east Asians have greater visceral fat and higher percentage body fat than other racial/ethnic groups^39^. These observations of decreased waist circumference and cortisol highlights that depression-related cortisol directs fat deposits viscerally^40^.

Our high retention rate is also quite noteworthy. We attribute this primarily to high level of skill of the community health educators who were carefully chosen, thoroughly trained, and regularly supervised. Participant satisfaction with the interventions was high^19^. Our culturally derived interventions were founded in culturally specific fundamentals about health, illness, symptoms, healers and cures. Rather than translating the DPP in Khmer, we started with Cambodian concepts, ideals, and collective history to address metabolic risk. The flexibility of the protocol for intervention delivery (rolling enrollment, makeup sessions) also facilitated high level of participation and retention.

### Limitations

Several limitations should be acknowledged. First, our sample was relatively small so we may not have been powered to detect changes between groups in secondary outcomes like hair cortisol and c-reactive protein. Our duration of follow-up was only 3 months after post-treatment and 15 months after baseline so the longer-term durability of these effects are unknown.

We did not have a sample size sufficient to investigate specific medication changes in the MTM group that might have led to lowered HbA1c. Resolution of drug therapy problems could involve medications being added/deleted, dose increased/decreased, switched to another medication, and/or adherence increased. Moreover, drug therapy problems and their resolution could pertain to any permutation of medications (e.g., statins, anti-retrovirals, steroids, antidepressants, etc.). A larger study with very granular data collection regarding MTM processes is necessary to understand what specific changes lead to a decrease in HbA1c. What is clear, however, is that in this randomized trial, the MTM intervention decreased HbA1c.

Because there is no objective measure of depressive symptoms, our assessment of depressive symptoms was per self-report which may be vulnerable to demand characteristics. Due to racial differences in the relationships between weight/BMI and diabetes for east Asians, we did not plan to examine weight or BMI as a primary outcome which may limit comparison to similar trials, though we do report values of weight and BMI at each timepoint. Our sample was geographically centered in New England where weather and neighborhood factors that affect lifestyle may not generalize to, say, southern California where the other large community of Cambodian Americans live.

## Conclusions

DREAM decreased biological (HbA1c) and behavioral (depressive symptoms) risk for type 2 diabetes, and did so in a challenging sample of non-English speaking, refugee participants. Similar approaches may apply to other communities with historical trauma and barriers to appropriate care. Even with adequate healthcare coverage through insurance or government healthcare, many immigrant and refugee groups will continue to have barriers to linguistically and culturally appropriate care. Many refugee and minority groups have developed CHE/CHW workforces and some have also developed their own culturally tailored diabetes prevention programs. Incorporating our cross-cultural and interdisciplinary model that incorporates medication therapy management may be beneficial to other minority, immigrant and refugee groups and other groups with high rates of depression.

## Data Availability

The datasets generated during and/or analyzed during the current study are available from the corresponding author on reasonable request.

## References

[1] Knol MJ, Twisk JW, Beekman AT, Heine RJ, Snoek FJ, Pouwer F (2006). Depression as a risk factor for the onset of type 2 diabetes mellitus A meta-analysis. Diabetologia.

[2] Mezuk B, Eaton WW, Albrecht S, Golden SH (2008). Depression and type 2 diabetes over the lifespan: A meta-analysis. Diabetes Care.

[3] Burke HM, Davis MC, Otte C, Mohr DC (2005). Depression and cortisol responses to psychological stress: A meta-analysis. Psychoneuroendocrinology.

[4] Valkanova V, Ebmeier KP, Allan CL (2013). CRP, IL-6 and depression: A systematic review and meta-analysis of longitudinal studies. J. Aff. Disord..

[5] Rotella F, Mannucci E (2013). Depression as a risk factor for diabetes: A meta-analysis of longitudinal studies. J. Clin. Psychiatry.

[6] Knowler WC, Barrett-Connor E, Fowler SE, Hamman RF, Lachin JM, Walker EA (2002). Reduction in the incidence of type 2 diabetes with lifestyle intervention or metformin. N. Engl. J. Med..

[7] Cezaretto A, Ferreira SR, Sharma S, Sadeghirad B, Kolahdooz F (2016). Impact of lifestyle interventions on depressive symptoms in individuals at-risk of, or with, type 2 diabetes mellitus: A systematic review and meta-analysis of randomized controlled trials. Nutr. Metab. Cardiovasc. Dis..

[8] Janney CA, Greenberg JM, Moin T, Kim HM, Holleman RG, Hughes M (2018). Does mental health influence weight loss in adults with prediabetes? Findings from the VA Diabetes Prevention Program. Gen. Hosp. Psychiatry.

[9] Schneider KL, Sullivan JC, Pagoto SL (2011). Translation of the diabetes prevention program into a community mental health organization for individuals with severe mental illness: A case study. Transl. Behav. Med..

[10] Marshall GN, Schell TL, Wong EC, Berthold SM, Hambarsoomian K, Elliott MN (2016). Diabetes and cardiovascular disease risk in Cambodian refugees. J. Immigr. Minor. Health.

[11] Marshall GN, Schell TL, Elliott MN, Berthold SM, Chun CA (2005). Mental health of Cambodian refugees 2 decades after resettlement in the United States. JAMA.

[12] Wagner JA, Bermudez-Millan A, Berthold SM, Buckley T, Buxton OM, Feinn R (2022). Risk factors for drug therapy problems among Cambodian Americans with complex needs: A cross-sectional, observational study. Health Psychol. Behav. Med..

[13] Polomoff CM, Bermudez-Millan A, Buckley T, Buxton OM, Feinn R, Kong S (2021). Pharmacists and community health workers improve medication-related process outcomes among Cambodian Americans with depression and risk for diabetes. J. Am. Pharm. Assoc..

[14] Peterman JN, Wilde PE, Liang S, Bermudez OI, Silka L, Rogers BL (2010). Relationship between past food deprivation and current dietary practices and weight status among Cambodian refugee women in Lowell, MA. Am. J. Public Health.

[15] Berthold SM, Loomis AM, Kuoch T, Scully M, Hin-McCormick MM, Casavant B (2019). Social disconnection as a risk factor for health among Cambodian refugees and their offspring in the United States. J. Immigr. Minor Health.

[16] Commission. APAA. *Needs assessment of Southeast Asian population in Connecticut*. 2014.

[17] Ackermann RT, O'Brien MJ (2020). Evidence and challenges for translation and population impact of the diabetes prevention program. Curr. Diab. Rep..

[18] Wagner J, Kong S, Kuoch T, Scully MF, Tan HK, Bermudez-Millan A (2015). Patient reported outcomes of 'Eat, Walk, Sleep': A cardiometabolic lifestyle program for Cambodian Americans delivered by community health workers. J. Health Care Poor Underserv..

[19] Wagner J, Bermudez-Millan A, Buckley T, Buxton OM, Feinn R, Kong S (2021). A randomized trial to decrease risk for diabetes among Cambodian Americans with depression: Intervention development, baseline characteristics and process outcomes. Contemp. Clin. Trials.

[20] Mollica RF, Wyshak G, de Marneffe D, Khuon F, Lavelle J (1987). Indochinese versions of the Hopkins Symptom Checklist-25: A screening instrument for the psychiatric care of refugees. Am. J. Psychiatry.

[21] Harris PA, Taylor R, Thielke R, Payne J, Gonzalez N, Conde JG (2009). Research electronic data capture (REDCap)—A metadata-driven methodology and workflow process for providing translational research informatics support. J. Biomed. Inform..

[22] Stout RL, Wirtz PW, Carbonari JP, Del Boca FK (1994). Ensuring balanced distribution of prognostic factors in treatment outcome research. J. Stud. Alcohol Suppl..

[23] Kuoch T, Scully M, Tan HK, Rajan TV, Wagner J (2014). The National Cambodian American Town Hall Meeting: A community dialogue on "eat, walk, sleep" for health. Prog. Community Health Partnersh..

[24] Hirshkowitz M, Whiton K, Albert SM, Alessi C, Bruni O, DonCarlos L (2015). National Sleep Foundation's sleep time duration recommendations: Methodology and results summary. Sleep Health.

[25] Excellence NIfHaC. *Preventing Type 2 Diabetes: Risk Identification and Interventions for Individuals at High Risk*. National Institute for Health and Clinical Excellence; 2012.

[26] Paulweber B, Valensi P, Lindström J, Lalic NM, Greaves CJ, McKee M (2010). A European evidence-based guideline for the prevention of type 2 diabetes. Horm. Metab. Res..

[27] Practitioners JCoP*. Pharmacists’ Patient Care Process*. May 29, 2014.

[28] Rhea JM, Molinaro R (2014). Pathology consultation on HbA(1c) methods and interferences. Am. J. Clin. Pathol..

[29] Matthews DR, Hosker JP, Rudenski AS, Naylor BA, Treacher DF, Turner RC (1985). Homeostasis model assessment: Insulin resistance and beta-cell function from fasting plasma glucose and insulin concentrations in man. Diabetologia.

[30] Bethancourt HJ, Ulrich MA, Almeida DM, Rosinger AY (2021). Household food insecurity, hair cortisol, and adiposity among Tsimane' Hunter–Forager–Horticulturalists in Bolivia. Obesity.

[31] Martin SS, Blaha MJ, Elshazly MB, Toth PP, Kwiterovich PO, Blumenthal RS (2013). Comparison of a novel method vs the Friedewald equation for estimating low-density lipoprotein cholesterol levels from the standard lipid profile. JAMA.

[32] Pickering TG, Hall JE, Appel LJ, Falkner BE, Graves J, Hill MN (2005). Recommendations for blood pressure measurement in humans and experimental animals: part 1: blood pressure measurement in humans: A statement for professionals from the Subcommittee of Professional and Public Education of the American Heart Association Council on High Blood Pressure Research. Circulation.

[33] Rosenthal R. *The Handbook of Research Synthesis*. In: Hedges HCaLV, editor. Russell Sage Foundation. 1994.

[34] Sun Y, You W, Almeida F, Estabrooks P, Davy B (2017). The effectiveness and cost of lifestyle interventions including nutrition education for diabetes prevention: A systematic review and meta-analysis. J. Acad. Nutr. Diet..

[35] Pan A, Sun Q, Okereke OI, Rexrode KM, Rubin RR, Lucas M (2012). Use of antidepressant medication and risk of type 2 diabetes: Results from three cohorts of US adults. Diabetologia.

[36] Price DW, Ma Y, Rubin RR, Perreault L, Bray GA, Marrero D (2013). Depression as a predictor of weight regain among successful weight losers in the diabetes prevention program. Diabetes Care.

[37] Cuijpers P, van Straten A, Warmerdam L (2007). Behavioral activation treatments of depression: A meta-analysis. Clin. Psychol. Rev..

[38] Kvam S, Kleppe CL, Nordhus IH, Hovland A (2016). Exercise as a treatment for depression: A meta-analysis. J. Aff. Disord..

[39] Deurenberg P, Deurenberg-Yap M, Guricci S (2002). Asians are different from Caucasians and from each other in their body mass index/body fat per cent relationship. Obes. Rev..

[40] Weber-Hamann B, Hentschel F, Kniest A, Deuschle M, Colla M, Lederbogen F (2002). Hypercortisolemic depression is associated with increased intra-abdominal fat. Psychosom. Med..

